# Traditional Chinese Medicine in Cancer Care: A Review of Controlled Clinical Studies Published in Chinese

**DOI:** 10.1371/journal.pone.0060338

**Published:** 2013-04-03

**Authors:** Xun Li, Guoyan Yang, Xinxue Li, Yan Zhang, Jingli Yang, Jiu Chang, Xiaoxuan Sun, Xiaoyun Zhou, Yu Guo, Yue Xu, Jianping Liu, Alan Bensoussan

**Affiliations:** 1 Centre for Evidence-based Chinese Medicine, Beijing University of Chinese Medicine, Beijing, China; 2 Centre for Complementary Medicine Research (CompleMED), University of Western Sydney, Sydney, New South Wales, Australia; 3 School of Preclinical Medicine, Beijing University of Chinese Medicine, Beijing, China; 4 School of Management, Beijing University of Chinese Medicine, Beijing, China; 5 School of Acupuncture and Moxibustion, Beijing University of Chinese Medicine, Beijing, China; 6 School of Humanities, Beijing University of Chinese Medicine, Beijing, China; Vanderbilt University Medical Center, United States of America

## Abstract

**Background:**

Traditional Chinese medicine (TCM) has been widely applied for cancer care in China. There have been a large number of controlled clinical studies published in Chinese literature, yet no systematic searching and analysis has been done. This study summarizes the current evidence of controlled clinical studies of TCM for cancer.

**Methods:**

We searched all the controlled clinical studies of TCM therapies for all kinds of cancers published in Chinese in four main Chinese electronic databases from their inception to November 2011. We bibliometrically analyzed the included studies and assessed the reporting quality.

**Results:**

A total of 2964 reports (involving 253,434 cancer patients) including 2385 randomized controlled trials and 579 non-randomized controlled studies were included. The top seven cancer types treated were lung cancer, liver cancer, stomach cancer, breast cancer, esophagus cancer, colorectal cancer and nasopharyngeal cancer by both study numbers and case numbers. The majority of studies (72%) applied TCM therapy combined with conventional treatment, whilst fewer (28%) applied only TCM therapy in the experimental groups. Herbal medicine was the most frequently applied TCM therapy (2677 studies, 90.32%). The most frequently reported outcome was clinical symptom improvement (1667 studies, 56.24%) followed by biomarker indices (1270 studies, 42.85%), quality of life (1129 studies, 38.09%), chemo/radiotherapy induced side effects (1094 studies, 36.91%), tumor size (869 studies, 29.32%) and safety (547 studies, 18.45%). Completeness and adequacy of reporting appeared to improve with time.

**Conclusions:**

Data from controlled clinical studies of TCM therapies in cancer treatment is substantial, and different therapies are applied either as monotherapy or in combination with conventional medicine. Reporting of controlled clinical studies should be improved based on the CONSORT and TREND Statements in future. Further studies should address the most frequently used TCM therapy for common cancers and outcome measures should address survival, relapse/metastasis and quality of life.

## Introduction

With an ageing worldwide population coupled with unhealthy lifestyles and increased medical intervention, the burden of disease and overall mortality has shifted gradually to primarily non-communicable diseases such as cardiovascular disease and cancer [1]. It is estimated that about 12.7 million cancer cases and 7.6 million cancer deaths occurred in 2008 [2], and the World Health Organization (WHO) estimates that 84 million people would die of cancer between 2005 and 2015 [3].

The earliest records of tumors can be traced back to inscriptions on bones and tortoiseshells in the 16th–11th century B.C., and the “malignant sores” with “swelling but without ulceration” recorded by traditional Chinese medicine (TCM) doctors in Qin Dynasty (221-207 B.C.) already presented various theories and approaches to treat cancer. [4].

TCM has increasingly become popular in the West including in cancer patients [5]. Chinese medicine plays an important role in minimizing disability, protecting cancer patients against suffering from complications, and helping patients to live well [6]. Chinese medicine may also assist in supportive and palliative care by reducing side-effects of conventional treatment or improving quality of life [7]. It is estimated the United States National Cancer Institute (NCI) spends around $120 million each year on CAM related research projects [8].

A recent review of surveys of complementary and alternative medicine use for cancer [9] identified 74 studies over the last 15 years, with 70% of publications occurring after 2005.

Previous reviews of TCM for cancer care in Chinese publications have identified 716 case reports involving 1,198 patients [10], and 1,217 case series reports involving 92,945 patients [11], which showed a large prevalence of a diversity of TCM clinical application for cancer patients. However, controlled clinical studies were not included in these two reviews. In order to catch a more comprehensive picture on TCM clinical usage for cancer care in China, we systematically reviewed Chinese literature to summarize the clinical evidence of controlled clinical studies in this area.

## Materials and Methods

### Literature Search

We searched four major Chinese electronic databases including China National Knowledge Infrastructure (CNKI) (1911-November 2011), Chinese Scientific Journal Database (VIP) (1989-November 2011), Chinese BioMedical Literature Database (CBM) (1978-November 2011), and Wanfang Database (1994-November 2011).

The Chinese searching terms were zhong yi (Chinese medicine), zhong yao (Chinese medicine/Chinese herbal medicine), zhong yi yao (Chinese medicine), zhong cheng yao (Chinese proprietary medicine), zhen (needling/acupuncture), jiu (moxibustion), tui na (tui na/massage), gua sha (scraping), ba guan (cupping), xue wei (acupoint), qi gong, min zu yao (ethnomedicine), min jian (folk); terms related to cancer disease including ai (cancer), liu (tumor), e (malignant), bai xue (leukemia), gu sui (bone marrow) and lin ba (lymph). Based on pilot searches, we noted improved outcomes by searching for any match with study citation, abstract, keyword or subject word.

### Study Selection

Randomized controlled trials (RCT) or non-randomized (clinical) clinical studies (CCS) with at least one group involving TCM treatment for all types of cancer-related patients including malignant tumor, malignant hematologic disease and patients with precancerous condition were included. Controlled studies reporting “random allocation” were regarded as RCT, while controlled studies without mentioning randomization were regarded as CCS, including non-randomized controlled clinical trial (CCT) and prospective/retrospective observational study.

Two authors (XL and GYY) screened the titles and abstracts of the hits from literature searching, and full papers were retrieved by downloading electronic versions (JC, JLY, XYZ, and YG) and hand searching when electronic versions were unavailable (GYY).

### Data Extraction

A structured data extraction form was designed (XL and XXL), and 10 authors (YZ, GYY, JLY, YG, XXS, XYZ, JC, XXL, YX and XL) participated in data extraction. The data extraction involved study information as followed:

We extracted bibliometric information, including citation information, publication types and funding information if available.

Clinical related indices consisted: 1) Disease and diagnosis: we recorded the cancer types according to International Statistical Classification of Diseases and Related Health Problems 10th Revision [12]. We extracted clinical-related conditions which the study addressed as the primary diagnosis of the included patients in the studies. Studies about prevention were specially categorized. Studies reported primary diagnosis related to TCM syndrome differentiation were specially recorded. We recorded the reporting completeness of diagnostic criteria, syndrome differentiation (bianzheng) and application of diagnostic gold standard (pathological diagnosis or cytological diagnosis). 2) Treatment and control: We distinguished conventional treatments and TCM, administration routes of TCM information both in treatment group and control group, further classification of herbal medicine and acupoint stimulation, and the reporting of treatment method, dosage and treatment duration for both TCM and conventional medicine. 3) Outcome measures: We extracted and classified clinical-related outcomes. If quality of life was reported, the tool for measuring was extracted if available. If the authors defined the graded effectiveness by combining a number of outcomes, for example, obvious improvement of quality of life and obvious reduction of tumor size meant “very effective” while obvious improvement of quality of life or obvious reduction of tumor size meant “effective”, rather than reporting each original outcome, we recorded this as “combined outcome”. Whether the author recommended the clinical application as the study report conclusion was also extracted.

We assessed the completeness of reporting according to CONSORT Statement [13] for RCTs and TREND Statement [14] for CCSs. We assessed the reporting of diagnosis criteria, inclusion/exclusion criteria, allocation methods, blinding, details of interventions and level of controls, and use of outcomes measures for quality of life. We identified whether the study was randomized and whether the method of randomization as reported by authors. We identified blinding as either to patients, physicians, evaluators and/or statisticians. If placebo was reported and “double blind” was mentioned, we classified the blinding as “blinding to physicians and patients”, while if placebo was reported without mentioning the details of blinding, we classified the blinding as “blinding to the patients”.

We summarised well designed (reporting both randomization methods and blinding details) studies reporting positive survival outcomes to identify any significant evidence for the TCM treatments.

All the extraction was verified by one author (XL), and any discrepancies were discussed with the other authors for consensus.

### Data Analysis

Data were presented by counts, percentage and frequency. Epidata (Version 13.02) was applied for data extraction, and Microsoft Excel and SPSS for Windows (version 17.0, Chicago, Illinois, USA) were applied for data analysis (XL, XXL, GYY).

## Results

### Bibliometric Information

**Figure 1:**
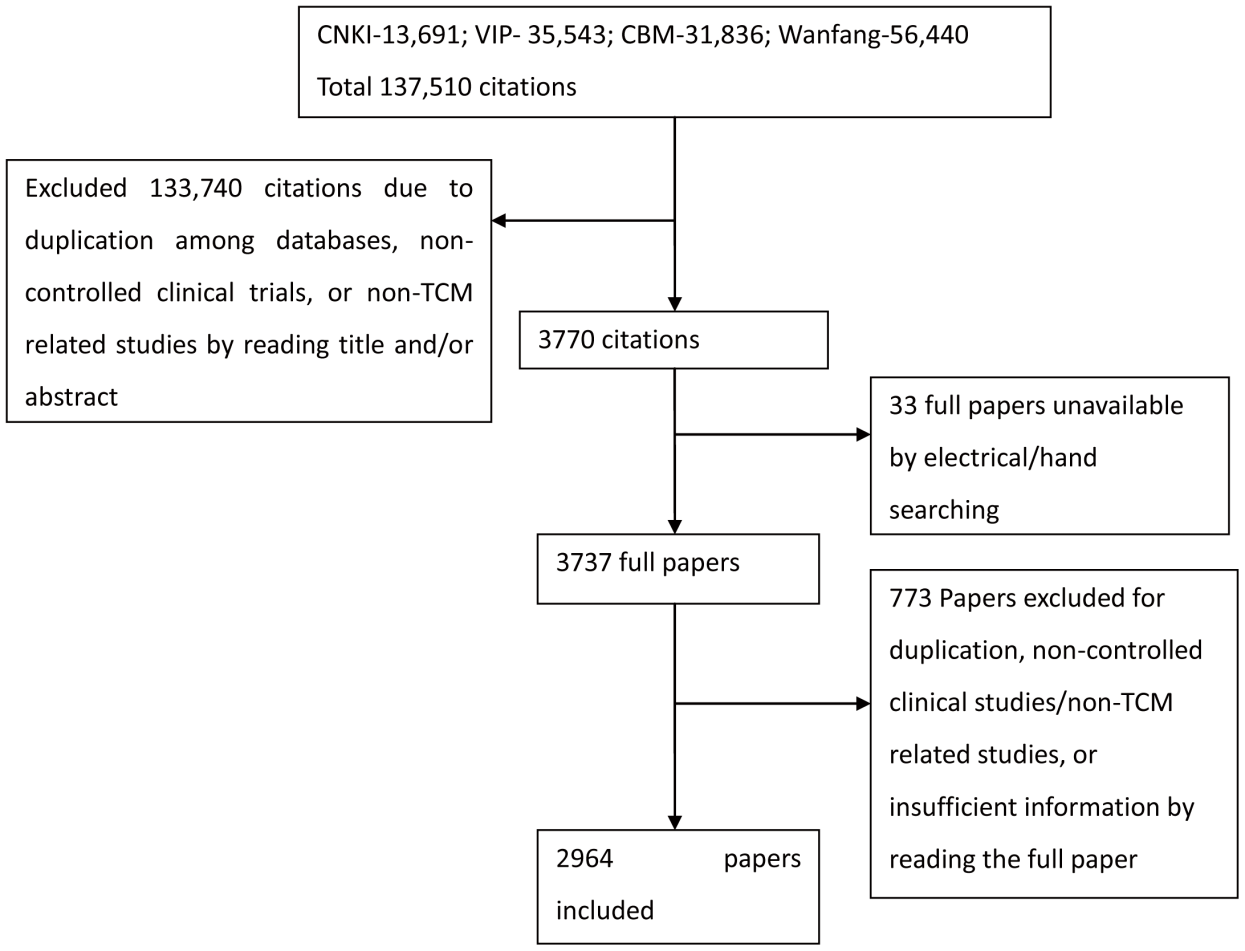
Flow chart of Literature searching and study selection. Presentation of the procedure of literature searching and study selection with numbers of articles at each stage.

A total of 137,510 citations were identified and screened based on searching in the four electronic databases, and 3737 full papers were retrieved. A final 2,964 articles involving 253,434 (10-1700, 85.50±70.59) participants were included after exclusion due to duplication, non-controlled clinical studies, non-cancer related studies or insufficient information (Figure 1).

**Figure 2:**
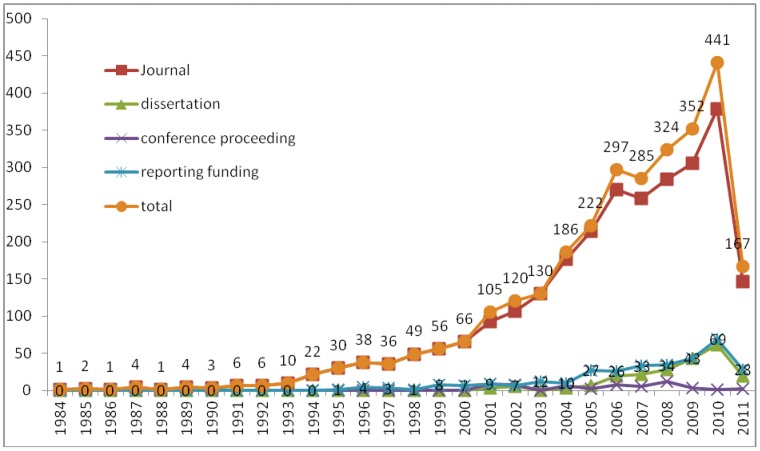
Publication number of controlled clinical studies of TCM for cancer published in Chinese. Presentation of the number of Chinese publication of controlled clinical studies of TCM for cancer in each year. The publication numbers of all the studies, journal articles, conference proceedings, dissertations, and the publications reporting funding information are shown respectively.

A total of 2385 RCTs and 579 CCSs were identified. The earliest controlled clinical study was a retrospective clinical report about integrative medicine treating esophageal cancer published in 1984 [15]. The earliest RCTs we identified were one study of herbal injection for cancer pain in 513 advanced-staged cancer patients [16] and another study of herbal decoction for bone marrow and immune function-related side effects induced by chemotherapy involving 40 patients [17], both of which were published in 1985. The quantity of publications increased especially after the year 2000. The publication types were further divided into journal articles, dissertations and conference proceedings. Studies reporting the funding information were specifically summarised in order to present the quantity change tendency, too. (Figure 2).

### Participants and Diseases

Among the 2964 controlled clinical studies, 1490 studies focused on the treatment of cancer, and the other studies focused on precancerous condition, cancer-related clinical conditions, post-surgery conditions and radio/chemotherapy induced side effects (Table S2). Leucopenia was the most frequently reported primary diagnosis in radio/chemotherapy-induced side effects (119 studies out of 991), and cancer pain showed the highest frequency in cancer-related conditions (123 studies out of 283).

Out of the total 2964 studies, 292 (9.85%) involving 26,585 (10.49%) patients focused on cancer or cancer-related conditions prevention. Prevention of relapse and/or metastasis took up 16 studies out of 17 for relapse and/or metastasis, occupying the largest percentage (94.12%) of prevention. There were also more studies related to the prevention of radiation injury (61.19%), and prevention of other drug induced side effects (60.00%) than the studies about treatment of the same clinical conditions. (Table S1).

**Figure 3:**
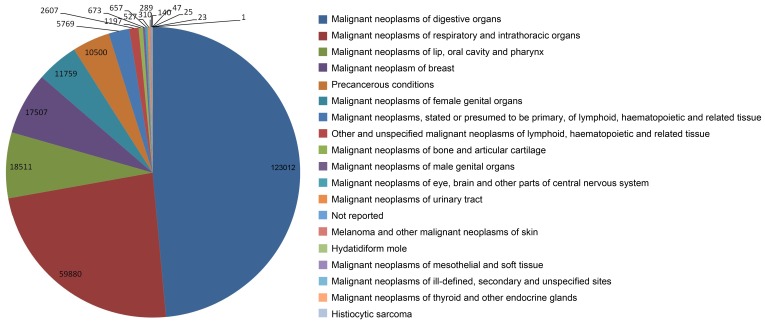
Cancer categories in controlled clinical studies of TCM for cancer published in China. Presentation of the categories of cancers reported in the include studies according to ICD-10.

**Figure 4:**
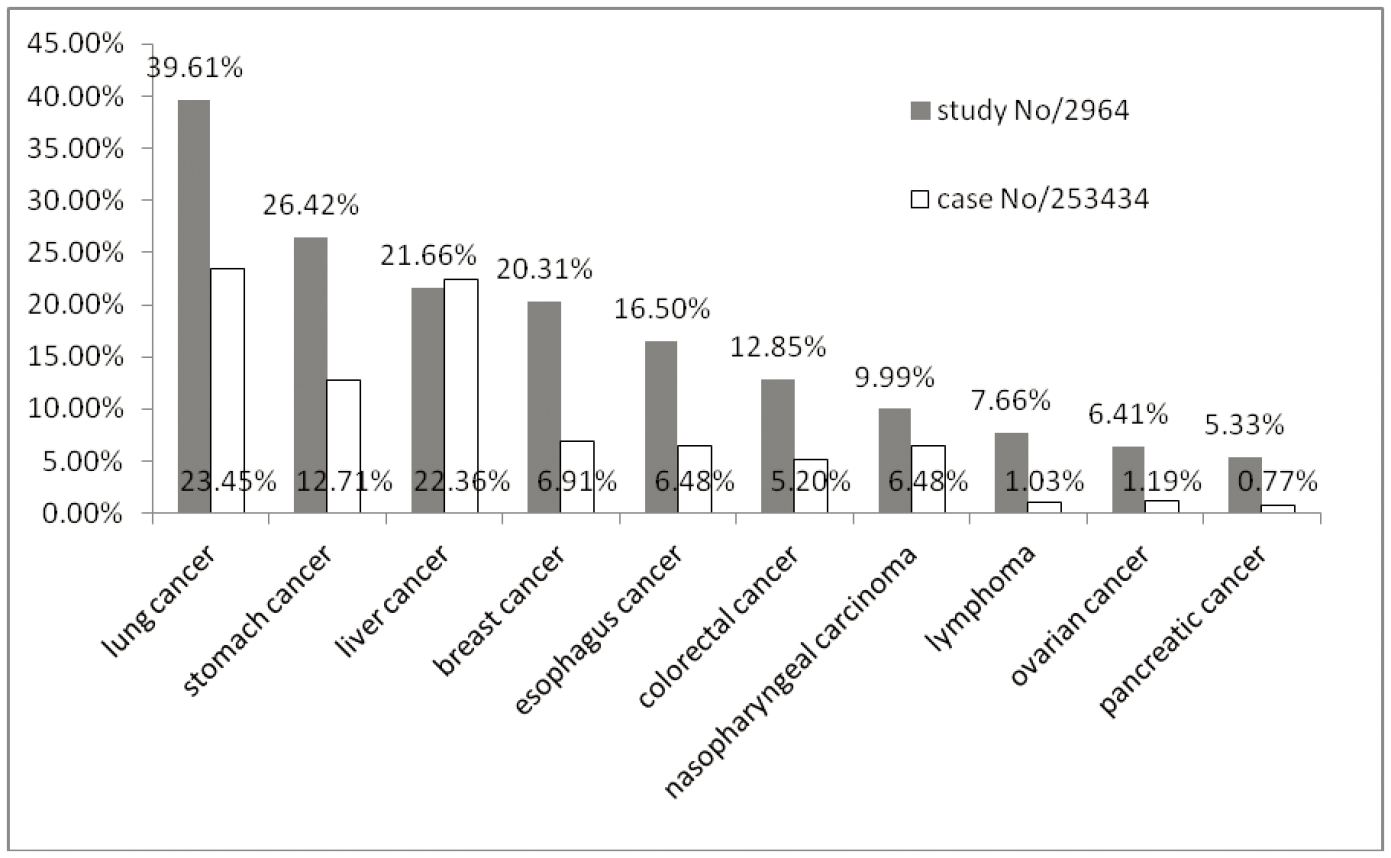
Top 10 cancer types with study numbers. Presentation of the top 10 most frequently reported cancer types calculate by study numbers. The numbers of participants ware also presented.

Cancer types were summarised into 17 categories according to ICD-10 (Table S2, Figure 3). Calculating by case numbers and study numbers, the highest prevalence cancer types treated, included in descending order of priority, were respectively lung cancer, liver cancer, stomach cancer, breast cancer, esophagus cancer, colorectal cancer, and nasopharyngeal cancer (Figure 4).

### Interventions in Different Groups

Among the total 2964 studies, the majority of 2770 (93.45%) studies reported studies with 2 arms, 175 (5.90%) studies reported 3 arm studies, while 19 (0.64%) reported 4 or more arms in one study.

Interventions in treatment groups involved purely TCM intervention in 836 (28.21%) studies, while TCM combined with conventional treatment in 2128 (71.79%) studies. The interventions in control groups involved purely conventional treatment in 2570 (86.71%) studies, TCM combined with conventional treatment in 204 (6.88%) studies, and purely TCM intervention in 182 (6.14%) studies.

#### TCM intervention

TCM interventions reported in the total 2964 studies were classified into herbal medicine, acupoint stimulation, dietary therapy, massage, TCM psychological intervention, TCM five element music therapy and qigong. Herbal medicine, including oral decoction, injection, external usage, perfusion, aerosol inhalation, mouth rinsing and nasal feeding, was the most frequently applied intervention in both treatment group (2667 studies, 89.98%) and control group (327 studies, 11.03%). Among all the types of herbal medicine, herbal medicine decoction was the majority. More than half (1145 out of 1720, 66.57%) decoctions were individualized, and most of the studies reported the detailed ingredients of the decoctions (Table 1). No study reported quality assessment of herbs or final herbal products.

**Table 1:** Intervention and control for cancer patients in controlled clinical studies of TCM for cancer published in Chinese.

Therapy	Treatment group	% (/2964)	Control group	% (/2964)
**Herbal medicine**	**2667**	**89.98%**	**327**	**11.03%**
oral medication	2098	70.78%	273	9.21%
decoction (reporting ingredients)	1720 (1631)	58.03%	149 (131)	5.03%
*individualized prescription (reporting ingredients)*	1145 (1089)	38.63%	103 (90)	3.48%
proprietary herbal products (reporting ingredients)	758 (564)	25.57%	185 (111)	6.24%
hospital prepared herbal medicine (reporting ingredients)	303 (278)	10.22%	22 (19)	0.74%
injection	450	15.18%	51	1.72%
external	200	6.75%	15	0.51%
perfusion	15	0.51%	1	0.03%
aerosol inhalation	5	0.17%	0	0.00%
mouth rinsing	5	0.17%	1	0.03%
nasal feeding	1	0.03%	0	0.00%
**Acupoint stimulation (acupoint composition)**	**379 (372)**	**12.79%**	**56 (50)**	**1.89%**
needling	131	4.42%	22	0.74%
acupoint injection	122	4.12%	20	0.67%
moxibustion	71	2.40%	5	0.17%
electronic acupuncture	44	1.48%	3	0.10%
acupoint application	28	0.94%	3	0.10%
ear acupuncture	18	0.61%	1	0.03%
acupressure	16	0.54%	0	0.00%
laser or microwave stimulation	4	0.13%	2	0.07%
cupping	2	0.07%	0	0.00%
acupoint nerve stimulation	2	0.07%	0	0.00%
bee-sting therapy	2	0.07%	0	0.00%
point embedding therapy	1	0.03%	0	0.00%
sham acupuncture (control group)			2	0.07%
**Other types of TCM intervention**	**31**	**1.05%**	**6**	**0.20%**
dietary therapy	15	0.51%	2	0.07%
massage	10	0.34%	3	0.10%
TCM psychological intervention	3	0.10%	0	0.00%
TCM five element music therapy	2	0.07%	0	0.00%
qigong	1	0.03%	1	0.03%
**Conventional medicine**				
chemotherapy	1170	39.47%	1328	44.80%
radiotherapy	354	11.94%	367	12.38%
western medicine or other routine treatment	622	20.99%	1228	41.43%
interventional therapy	162	5.47%	155	5.23%
surgery	73	2.46%	68	2.29%
**Reporting completeness of interventions**				
no information about medication duration	410	13.83%	376	12.69%
no information about dosage	133	4.49%	190	6.41%
no information about medication	38	1.28%	114	3.85%

Out of 379 (12.79%) and 56 (1.89%) studies reporting acupoint stimulation as the TCM treatment in treatment groups and control groups respectively, needling acupuncture and acupoint injection were dominating. Most of the studies related to acupoint stimulation reported acupoint composition (treatment group 372 studies, 86.28%, control group 50 studies, 89.29%) (Table 1).

#### Conventional treatment

Conventional treatment reported in the 2964 studies included chemotherapy, Western medicine as routine treatment, radiotherapy, interventional therapy and surgery. Chemotherapy was the dominating conventional treatment in both treatment group (1170 studies, 54.98% in the 2128 studies reporting TCM+conventional medicine) and control group (1328 studies, 47.87% in the 2774 studies reporting TCM+conventional medicine or conventional medicine only), followed by western medicine or other routine treatments (Table 1).

#### Control types

There were 25 studies applied placebo and 2 applied sham acupuncture as the control, and another 6 studies did not give any treatment to the patients in the control groups.

#### Detailed information of the intervention

Most studies reported the details of treatment such as method description, dosage and treatment duration. There were 410 (13.83%) studies failed to present any treatment information for treatment group and 376 (12.69%) for control group (Table 1).

### Outcome Measurement

The most-frequently reported outcome measurement was clinical symptom (1667 studies, 56.24%), followed by laboratory indices (1270 studies, 42.85%), quality of life (1129 studies, 38.09%), chemo/radiotherapy induced side effects (1094, 36.91%), tumor size (869 studies, 29.32%) and safety (547 studies, 18.45%). Survival was reported in 433 (14.61%) studies while metastasis and relapse were reported in 109 (3.68%) and 101 (3.41%). Among the total 1129 studies reporting quality of life as the outcome measurement, Karnofsky Score was applied in most of the studies (942 studies, 83.44%). There were 41 studies reported quality of life measured by “QOL Score” without giving any other detailed information, and we could not identify the exact tool by any of the information provided by the publication and references. (Table 2).

**Table 2:** Outcome measurements reported in controlled clinical studies of TCM for cancer published in Chinese.

Outcome measurement		No.	Frequency(/2964)
clinical symptom		1667	56.24%
biomarker indices		1270	42.85%
quality of life		1129	38.09%
	Karnofsky Score	942	31.78%
	EORTC-QLQ	58	1.96%
	QOL score without specific information	41	1.38%
	FACT	27	0.91%
	ECOG Score	5	0.17%
	WHOQOL	5	0.17%
	SF-36	4	0.13%
	self-description	3	0.10%
	FLIC Scale	2	0.07%
	GQLI (Gastrointestinal Quality of Life Index)	2	0.07%
	TCM quality of life scale	2	0.07%
	other	15	0.51%
	not reported	27	0.91%
chemo/radiotherapy induced side effects		1094	36.91%
tumor size		869	29.32%
safety		547	18.45%
survival		433	14.61%
post-surgery side effects		156	5.26%
body weight		126	4.25%
metastasis		109	3.68%
relapse		101	3.41%
pain		71	2.40%
imaging indexes		47	1.59%
fever		29	0.98%
depression indexes		10	0.34%
appetite		8	0.27%
economic indexes		6	0.20%

There were totally 516 studies (17.41%) reported clinical effectiveness levels graded by several outcomes together without giving adequate information for each of the original outcome. (Table 2).

### Overall Recommendation of Treatments

Among the total 2964 studies, 756 (25.51%) studies recommended generalizing the treatment to the broader community based on treatment effectiveness. Commonly seen recommendations included: “this treatment method is very effective with good safety, and is very suitable for generalization in the clinical applications”.

### Completeness of Reporting

Out of 2964 studies, 2136 (72.06%) reported diagnosis criteria of the included patients, 2315 (78.10%) reported golden standard for cancer diagnosis, such as pathological diagnosis or cytologic diagnosis, 1020 (34.41%) reported inclusion and/or exclusion criteria for the participants, while 1339 (45.18%) reported the stages of cancer.

The reporting rates across different publication types were different in diagnosis criteria, golden standard of cancer, inclusion/exclusion criteria, cancer staging and randomization method. Dissertations more frequently reported all of the detailed information mentioned above. (Table 3, Table 4).

**Table 3:** Completeness of Reporting across different types of publications.

	Total No. ofthe studies	Reportingfunding	*Impact of reporting with* *or without funding* *reporting (p value) Δ*	Journal articles	Dissertation	*Compared with journal* *article (chi-square,* *p value)*	C onference proceeding	*Compared with journal article (chi-square,* *p value)*
**Total**	2964	322		2698	209		57	
**Diagnosis criteria**	2136	242	*0.191*	1895	201	*64.863, <0.001* *	40	*<0.001, 0.992*
**Golden standard**	2315	251	*0.944*	2082	186	*15.821, <0.001* *	47	*0.889, 0.425*
**Inclusion/exclusion Criteria**	1020	158	*<0.001* * *(OR = 1.99)*	789	204	*403.083, <0.001* *	27	*8.796, 0.003* *
**Staging**	1339	169	*0.005* * *(OR = 1.31)*	1152	154	*75.271, <0.001* *	33	*5.295, 0.021*

* : statistically significant difference.

Δ: based on logistic regression.

**Table 4:** Overall picture of methodology quality of RCT and CCS of TCM for cancer.

	Total No. of the studies	Reporting funding	*Impact of reporting with or without funding reporting* *(p value) Δ*	Journal articles	Dissertation	*Compared with journal* *article (chi-square,* *p value)*	Conference proceeding	*Compared with journal article (chi-square value, p value)*
**Total**	2964	322		2698	209		57	
**RCT**	2385	289		2191	157		37	
**Randomization method**	394	95	*<0.001* * *(OR = 2.94)*	326	58	*52.134, <0.001* *	10	*4.193, 0.041*
**Blinding**	63	17		53	9		1	
**Who is blinded**	40	14	*0.069*	34	5	*0.894,0.819* **	1	*0.894,0.819* **

* statistically significant difference.

** p value of chi-square across journal articles, dissertation and conference proceeding.

Δ: based on logistic regression.

Studies with funding information more frequently reported inclusion/exclusion criteria, cancer staging and blinding details than those without funding information. (Table 3, Table 4).

**Figure 5:**
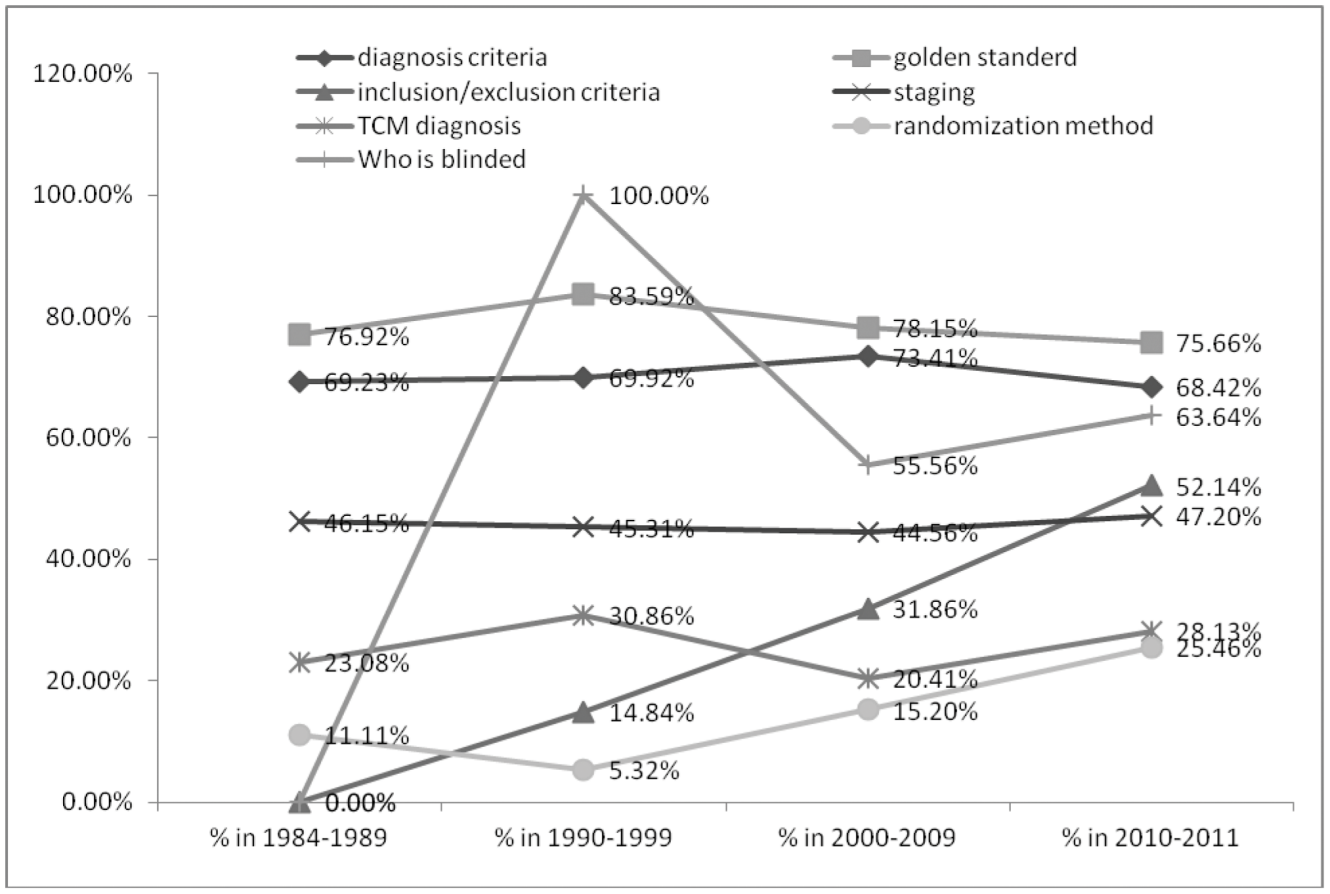
Reporting completeness, randomization methods and blinding across years. Percentage of reporting diagnosis criteria, inclusion/exclusion criteria, TCM diagnosis, golden standard of cancer, cancer staging, randomization method and blinding in the time order. Years are presented by the clusters of 1984–1989, 1990–1999, 2000–2009 and 2010–2011.

Reporting of inclusion/exclusion criteria, randomization method and details of blinding increased over time. (Figure 5).

There were 679 (22.91%) studies reported TCM diagnosis based on syndrome differentiation as diagnosis standards as selection criteria, reference for treatment or for outcome measuring.

#### Randomization

Among the 2385 RCTs, 394 (16.52%) reported adequate randomization methods, such as random table, computer software randomization, drawing lots and throwing coin. There were 33 studies reported “envelope method” or “random envelop “as the randomization method, and we counted them as adequate randomization method, too. Conference proceedings and dissertations had higher rate of reporting randomization methods out of studies mentioning “randomization” (Table 4).

#### Blinding

A total of 63 studies reported blinding to the participants, and 40 studies out of them (63.49%) reported the people who were blinded, such as participants, physicians, outcome measurers and statisticians. The other 23 studies just mentioned “blinding”, “single blinding” or “double blinding” without providing any further information. Again, conference proceeding and dissertations reported the people who were blinded more frequently if “blinding” was mentioned (Table 4).

### Significant Evidence of TCM Treatments

There were five studies [18]–[22] reporting relatively well designed RCTs with positive survival findings using Chinese herbal medicine. One study used herbal extract granules, another used a herbal capsule, and 3 used conventional Chinese herbal decoctions. Two of the studies reported TCM alone as the treatment intervention and the other 3 involved integrative TCM and conventional medicine. (Table S3).

## Discussion

This study systematically identified and analyzed controlled clinical studies of TCM for cancer, and included 2964 studies from 1984 to 2011. This is the continuous part of previous reviews on case reports and case series [10], [11] with the same topic. Unlike case reports and case series, Chinese publications of controlled clinical studies of TCM for cancer did not commence until 1984. This was consistent with the starting time of clinical trials in China, as a previous searching found that the earliest clinical trial in China started in the end of 1970s, and the first clinical trial in TCM area was a study of Chinese herbal injection for angina published in 1983 [23]. The number of publications increased with time, especially after 2000, which paralleled significant development of controlled clinical studies in China, and also possibly of the establishment of Chinese medical databases. Publication numbers fall in 2011 which may be partially due to the time lag in recording of trials by the databases.

The most frequently reported cancer types were consistent with the most common types of cancer (lung, stomach, liver, colon and breast cancer) causing deaths each year according to WHO [24] and basically consistent with the estimation of the new cancer diagnosis for developing countries (lung & bronchus cancer, stomach cancer, liver cancer, breast cancer and colon & rectum cancer) [2] Leukemia was ranked 10^th^ as calculated by patient cases numbers, and was not ranked within the top 10 cancer types if calculated by study numbers, which was quite different from case series (5th rank) and case reports (2nd rank). One possibility is that the treatment of leukemia is quite individualized, and there tends to be more publications of case series and case reports where more flexible and variable treatments are allowed than strict standardized interventions expected in RCTs. Both clinical observations and controlled clinical trials should be implemented to confirm the effectiveness and safety in the future.

The clinical application of TCM is of high clinical interest not only in the treatment of cancer and cancer related conditions, but also in its prevention. We identified 292 studies involving 26,585 patients using TCM treatment for prevention of relapse/metastasis of cancer or other cancer related conditions. Compared to treatment interventions, TCM seems to have higher impact in the prevention of relapse and/or metastasis, hemorrhage, radiotherapy induced inflammation, radiation injury, chemo/radiotherapy induced nausea and/or vomiting or other gastrointestinal disorder, other chemotherapy included side effects, and other drug-induced side effects. This is consistent with previously identified role of TCM as complementary medicine adjuvant or postal to conventional treatment [6]–[7], [9].

In our literature search, whilst we did not apply any terms directly related to precancerous lesions; we nevertheless identified 98 studies involving 11,759 patients with precancerous conditions. A more comprehensive search with more precise search terms including specific names of each precancerous condition should be implemented for a complete view of studies of TCM in cancer prevention.

Reporting completeness, including recording of inclusion/exclusion criteria, randomization method for RCTs, and recording of blinding improved with time. However, around half of the studies still did not provide adequate information around patient diagnosis and recruitment. This could be directly due to the poor reporting of studies, or indeed that some of these studies were actually retrospective clinical records summary instead of prospective clinical trials although the published article claimed itself as “clinical trial” or “clinical study”. It is necessary and essential for a prospective clinical study to recruit participants based on pre-defined standardized diagnosis criteria and clear selection criteria.

As far as the methodology is concerned, according to CONSORT Statement [13], a randomized controlled trial must report the details of randomization methods and blinding if blinding was implemented, and for non-randomized clinical study, TREND (Improving the Reporting Quality of Nonrandomized Evaluations of Behavioral and Public Health Interventions) Statement [14] requires that the authors report whether or not participants, those administering the interventions, and those assessing the outcomes were blinded to study condition assignment. We recommend that future publications give detailed information of randomization methods including sequence generation and randomization concealment and blinding information including whether blinding is used and who are blinded if available. If the report is based on existing previous data, the article should clearly articulate that it is a retrospective report so as todistinguish itself from prospective clinical studies.

If TCM syndrome differentiation is applied, the standard for judgment should be clearly stated.

Quality of life as one of the most important clinical outcomes and closely related to the patient wellbeing and increasingly included in studies. However the tools for measuring quality of life should have been more thoroughly reported with standard references in order to ensure clearer contribution to clinical decision making and design of further studies.

Funded studies, conference proceedings and dissertations seemed to generate higher report quality, which indicates that peer reviews is important for reporting of clinical studies, and the researchers in China are aware and able to do better research, however many of the Chinese journals haven’t paid enough attention to the standard and strict requirement when accepting submissions. It is urgently suggested that journals in China endorse international standards such as CONSORT for randomized controlled trials and TREND for non-randomized controlled trials in their author guidelines to improve the quality of clinical studies and reporting.

More than 20% of the studies failed to provide adequate gold standard diagnosis for cancers, which could creates risks in interpretation of data. As an essential element of cancer studies, we suggest every clinical studyreport clear methods of cancer diagnosis in the future. We searched the Chinese publications based on general searching terms for example zhong yi (Chinese medicine) and zhong yao (Chinese herbal medicine), publications only labeled by the exact treatment such like the name of the herbal formula would have been missed out. More standard labelling is responsible for a more complete searching. We recommend that the future publication have at least one label related to Chinese medicine or herbal medicine for keyword or subject word if the study is TCM-related.

It was difficult to judge whether papers were reports of a prospective clinical studies without adequate information such as randomization methods and inclusion/exclusion criteria. Thus we combined together all clinical studies which involved comparison between different groups of patients receiving different interventions. Whilst most of studies mentioned “randomization”, we could not assess more than 2,000 studies for study type (retrospective or prospective), and could not apply strict methodological evaluation as described in Cochrane Handbook [25]. Future studies should focus on specific types of TCM treatment for certain cancer with more detailed evaluation and analysis to ensure conclusions around effectiveness and safety and contribute to evidence-based clinical recommendations.

With regards to reviews of case series and case reports of TCM for cancer, individualized Chinese herbal medicine was the most frequently reported intervention. We suggest that the future studies focus on tailoring of treatment in cancer care.

Among the studies published in Chinese, herbal medicine is the main TCM intervention for cancer, comprising almost 90% in the total 2964 studies, which is also 7 times the number of acupuncture studies. This difference is much larger compared to a review based on English publications (acupuncture in 71 studies while Chinese herbal medicine in 11 studies) [9], which might reflect policy and levels of use of herbal medicine in different countries as well as publication bias. Although most of the included studies involved herbal medicine, there is no study reporting quality assessment of herbs or herbal products. Since the quality of herbal medicine is closely related to the clinical effectiveness and safety, and also essential for the quality of clinical study [26], this issue should be addressed in the future.

There have been a large number of controlled clinical studies of TCM for cancer, especially certain types with higher frequency such as lung cancer, liver cancer, stomach cancer, colorectal cancer, breast cancer, esophagus cancer, nasopharyngeal cancer, cervical cancer, ovarian cancer and pancreatic cancer. Systematic reviews could be implemented in order to access the effectiveness and safety of the TCM treatments for thesis types of cancers so that higher quality of evidence could be brought about based on the existing clinical information, the money and time have been spent, for the contribution to the clinical practice. One literature review of systematic reviews of TCM published in PubMed and Cochrane Library during the year 1999 to 2009 [26] identified 4 systematic reviews of TCM for cancer care including nasopharyngeal cancer, esophageal cancer, unrespectable hepatocellular cancer, and cancer pain respectively. We also identified Cochrane systematic reviews or protocols about Chinese herbal medicine for certain types of cancer including gastric precancerous lesions [27], Chinese herbal medicine for advanced pancreatic cancer [28], Chinese herbal medicine for chemotherapy side effects in colorectal cancer [29], Chinese herbal medicine for side effects of breast cancer [30], and herbal medicine for advanced colorectal cancer [31].

### Conclusions

In future standard and adequate reporting of TCM cancer studies is essential. More attention should be paid to reporting clinical outcomes of importance to patient care and clinical decision making including survival times, extent of relapse/metastasis and quality of life.

## Supporting Information

Table S1 **Cancer-related clinical condition of the cancer patients treated by TCM in the controlled clinical studies.** The study numbers and case numbers of cancer-related clinical conditions of the cancer patients treated by TCM. The clinical diagnosis is further classified as treated by TCM and prevented by TCM.
(DOC)

Table S2 **Types of cancer treated by TCM in controlled clinical studies published in Chinese journals.** Types of cancer treated by TCM in controlled clinical studies according to the category of. ICD-10 codes of all the cancers are presented in the table.
(DOC)

Table S3 **Characteristics of studies with significant evidence of TCM treatments for cancer published in Chinese.** The characteristics of 5 RCTs of TCM for cancer reporting randomization methods and blinding information with outcomes of survival, relapse and/or metastasis and quality of life.
(DOC)
