# Correction: Traditional Chinese Medicine in Cancer Care: A Review of Controlled Clinical Studies Published in Chinese

**DOI:** 10.1371/annotation/b53a0b8b-3eb6-44a2-9c37-bc9bb66bfe7e

**Published:** 2013-06-27

**Authors:** Xun Li, Guoyan Yang, Xinxue Li, Yan Zhang, Jingli Yang, Jiu Chang, Xiaoxuan Sun, Xiaoyun Zhou, Yu Guo, Yue Xu, Jianping Liu, Alan Bensoussan

The number of the third listed grant in the Funding statement is incorrect. The correct grant number is: 201207007. 

