# Supplementary material for: Traditional Chinese Medicine in Cancer Care: A Review of Controlled Clinical Studies Published in Chinese
Source: PLoS One. 2013 Apr 3;8(4):e60338. doi: 10.1371/journal.pone.0060338 (PMC3616129; doi:10.1371/journal.pone.0060338)
Supplement: Table S1 — Cancer-related clinical condition of the cancer patients treated by TCM in the controlled clinical studies. The study numbers and case numbers of cancer-related clinical conditions of the cancer patients treated by TCM. The clinical diagnosis is further classified as treated by TCM and prevented by TCM. (DOC) [file pone.0060338.s001.doc]

Table S1 Cancer-related clinical condition of the cancer patients treated by TCM in the controlled clinical studies

| **clinical condition** | **study No. (n1)** | **frequency (/2964)** | **case No. (n2)** | **frequency (/253434)** | **Study No. of prevention** | **% of prevention (/n1)** | **case No. of prevention** | **% of prevention (/n2)** |
| --- | --- | --- | --- | --- | --- | --- | --- | --- |
| cancer | 1406 | 47.44% | 120929 | 47.72% | 0 | 0.00% | 0 | 0.00% |
| metastasis cancer | 49 | 1.65% | 3674 | 1.45% | 9 | 18.37% | 726 | 19.76% |
| relapse and/or metastasis | 17 | 0.57% | 1862 | 0.73% | 16 | 94.12% | 1706 | 91.62% |
| relapse cancer | 12 | 0.40% | 848 | 0.33% | 4 | 33.33% | 388 | 45.75% |
| **subtotal** | **1484** | **50.07%** | **127313** | **50.24%** | **29** | 1.95% | **2820** | 2.22% |
| precancerous condition | 98 | 3.31% | 11759 | 4.64% | 3 | 3.06% | 430 | 3.66% |
| **subtotal** | **98** | **3.31%** | **11759** | **4.64%** | **3** | 3.06% | **430** | 3.66% |
| pain | 123 | 4.15% | 10524 | 4.15% | 1 | 0.81% | 94 | 0.89% |
| malignant effusion | 70 | 2.36% | 4241 | 1.67% | 0 | 0.00% | 0 | 0.00% |
| fever | 30 | 1.01% | 2066 | 0.82% | 0 | 0.00% | 0 | 0.00% |
| depression or other emotional disorder | 14 | 0.47% | 1205 | 0.48% | 0 | 0.00% | 0 | 0.00% |
| infection | 11 | 0.37% | 1004 | 0.40% | 3 | 27.27% | 260 | 25.90% |
| cancer complications | 9 | 0.30% | 815 | 0.32% | 0 | 0.00% | 0 | 0.00% |
| cachexia | 8 | 0.27% | 463 | 0.18% | 0 | 0.00% | 0 | 0.00% |
| anemia | 6 | 0.20% | 382 | 0.15% | 0 | 0.00% | 0 | 0.00% |
| fatigue | 6 | 0.20% | 507 | 0.20% | 0 | 0.00% | 0 | 0.00% |
| hemorrhage | 2 | 0.07% | 111 | 0.04% | 1 | 50.00% | 60 | 54.05% |
| hyperhidrosis | 2 | 0.07% | 158 | 0.06% | 0 | 0.00% | 0 | 0.00% |
| hot flash | 1 | 0.03% | 73 | 0.03% | 0 | 0.00% | 0 | 0.00% |
| **subtotal** | **282** | **9.51%** | **21549** | **8.50%** | **5** | 1.77% | **414** | 1.92% |
| post-surgery urinary retention | 20 | 0.67% | 1368 | 0.54% | 3 | 15.00% | 230 | 16.81% |
| post-surgery urinary disorder | 13 | 0.44% | 1378 | 0.54% | 0 | 0.00% | 0 | 0.00% |
| post-surgery upper limb edema | 10 | 0.34% | 2028 | 0.80% | 0 | 0.00% | 0 | 0.00% |
| post-surgery intestinal obstruction | 7 | 0.24% | 364 | 0.14% | 0 | 0.00% | 0 | 0.00% |
| post-surgery abdominal distension | 4 | 0.13% | 226 | 0.09% | 0 | 0.00% | 0 | 0.00% |
| post-surgery gastroparesis | 2 | 2.40% | 71 | 0.00% | 0 | 0.00% | 0 | 0.00% |
| other post-surgery conditions | 46 | 1.55% | 3572 | 1.41% | 13 | 28.26% | 1280 | 35.83% |
| **subtotal** | **102** | **5.77%** | **9007** | **3.53%** | **16** | 15.69% | **1510** | 16.76% |
| Leukopenia | 119 | 4.01% | 11597 | 4.58% | 11 | 9.24% | 859 | 7.41% |
| radiotherapy induced inflammation | 110 | 3.71% | 9765 | 3.85% | 38 | 34.55% | 3835 | 39.27% |
| immune function related laboratory indexes | 108 | 3.64% | 7388 | 2.92% | 1 | 0.93% | 40 | 0.54% |
| nausea and/or vomiting | 96 | 3.24% | 7805 | 3.08% | 48 | 50.00% | 3774 | 48.35% |
| gastrointestinal disorder | 89 | 3.00% | 7543 | 2.98% | 25 | 28.09% | 2078 | 27.55% |
| other laboratory indexes | 85 | 2.87% | 5811 | 2.29% | 0 | 0.00% | 0 | 0.00% |
| other chemotherapy induced side effects | 140 | 4.72% | 11613 | 4.58% | 42 | 30.00% | 4084 | 35.17% |
| radiation injury | 67 | 2.26% | 5677 | 2.24% | 41 | 61.19% | 3352 | 59.05% |
| other radiotherapy induced side effects | 38 | 1.28% | 3745 | 1.48% | 12 | 31.58% | 1458 | 38.93% |
| myelosuppression | 38 | 1.28% | 3367 | 1.33% | 6 | 15.79% | 424 | 12.59% |
| hiccup | 27 | 0.91% | 1767 | 0.70% | 1 | 3.70% | 102 | 5.77% |
| ascites | 24 | 0.81% | 1397 | 0.55% | 0 | 0.00% | 0 | 0.00% |
| other chemo/radiotherapy induced side effects | 15 | 0.51% | 1668 | 0.66% | 4 | 26.67% | 403 | 24.16% |
| constipation or bowel passing disorder | 11 | 0.37% | 1357 | 0.54% | 2 | 18.18% | 227 | 16.73% |
| diarrhea | 11 | 0.37% | 786 | 0.31% | 0 | 0.00% | 0 | 0.00% |
| chemotherapy induced oral ulcer | 8 | 0.27% | 930 | 0.37% | 3 | 37.50% | 414 | 44.52% |
| radiotherapy induced dry mouth | 7 | 0.24% | 422 | 0.17% | 2 | 28.57% | 122 | 28.91% |
| other drug-induced side effects | 5 | 0.17% | 355 | 0.14% | 3 | 60.00% | 239 | 67.32% |
| **subtotal** | **998** | **33.67%** | **82993** | **32.75%** | **239** | 23.95% | **21411** | 25.80% |
| **total** | **2964** |  | **253434** |  | **292** | 9.85% | **26585** | 10.49% |
