# Supplementary material for: Traditional Chinese Medicine in Cancer Care: A Review of Controlled Clinical Studies Published in Chinese
Source: PLoS One. 2013 Apr 3;8(4):e60338. doi: 10.1371/journal.pone.0060338 (PMC3616129; doi:10.1371/journal.pone.0060338)
Supplement: Table S2 — Types of cancer treated by TCM in controlled clinical studies published in Chinese journals. Types of cancer treated by TCM in controlled clinical studies according to the category of. ICD-10 codes of all the cancers are presented in the table. (DOC) [file pone.0060338.s002.doc]

Table S2 Types of cancer treated by TCM in controlled clinical studies published in Chinese journals

| **ICD-10** | **cancer name** | **Study No.** | **% (/2964)** | **Case No.** | **% (/253434)** |
| --- | --- | --- | --- | --- | --- |
| **Malignant neoplasms of lip, oral cavity and pharynx (C00-C14)** | |  |  |  |  |
| C00 Malignant neoplasm of lip | lip carcinoma | 1 | 0.0337% | 2 | 0.0008% |
| C00.4 Lower lip, inner aspect | buccal carcinoma | 5 | 0.1687% | 14 | 0.0055% |
| C02.9 Tongue, unspecified | tongue carcinoma | 14 | 0.4723% | 413 | 0.1630% |
| C03 Malignant neoplasm of gum | gum carcinoma | 7 | 0.2362% | 28 | 0.0110% |
| C05 Malignant neoplasm of palate | mandible carcinoma | 3 | 0.1012% | 17 | 0.0067% |
| C05.1 Soft palate | carcinoma of soft palate | 2 | 0.0675% | 11 | 0.0043% |
| C06.0 Cheek mucosa | buccal mucosa carcinoma | 3 | 0.1012% | 8 | 0.0032% |
| C06.9 Mouth, unspecified | oral carcinoma | 9 | 0.3036% | 931 | 0.3674% |
| C07 Malignant neoplasm of parotid gland | parotid carcinoma | 4 | 0.1350% | 15 | 0.0059% |
| C08.0 Submandibular gland | submandibular gland carcinoma | 2 | 0.0675% | 2 | 0.0008% |
| C09 Malignant neoplasm of tonsil | tonsillar carcinoma | 9 | 0.3036% | 65 | 0.0256% |
| C10 Malignant neoplasm of oropharynx | maxilla carcinoma | 1 | 0.0337% | 4 | 0.0016% |
| C11 Malignant neoplasm of nasopharynx | nasopharyngeal carcinoma（including nasal carcinoma and nasal malignant granuloma） | 296 | 9.9865% | 16416 | 6.4774% |
| C14.0 Pharynx, unspecified | pharyngeal cancer | 5 | 0.1687% | 12 | 0.0047% |
| C07-C14,C32-33 malignant neoplasm of head and neck | head and neck cancer | 13 | 0.4386% | 573 | 0.2261% |
| **Malignant neoplasms of digestive organs (C15-C26)** | |  |  |  |  |
| C15.9 Oesophagus, unspecified | esophagus cancer | 489 | 16.4980% | 16428 | 6.4822% |
| C16.0 Cardia | cardiac carcinoma | 47 | 1.5857% | 1053 | 0.4155% |
| C16.9 Stomach, unspecified | stomach cancer | 783 | 26.4170% | 32222 | 12.7142% |
| C17.0 Duodenum | duodenal carcinoma | 3 | 0.1012% | 16 | 0.0063% |
| C17.0 Duodenum | ampullary carcinoma | 3 | 0.1012% | 8 | 0.0032% |
| C17 Malignant neoplasm of small intestine | small intestinal carcinoma | 1 | 0.0337% | 3 | 0.0012% |
| C22.1 Intrahepatic bile duct carcinoma | cholangiocarcinoma | 24 | 0.8097% | 93 | 0.0367% |
| C22.9 Liver, unspecified | liver cancer | 642 | 21.6599% | 56664 | 22.3585% |
| C23 Malignant neoplasm of gallbladder | gallbladder carcinoma | 19 | 0.6410% | 156 | 0.0616% |
| C24.9 Biliary tract, unspecified | biliary tract cancers | 1 | 0.0337% | not reported |  |
| C25.9 Pancreas, unspecified | pancreatic cancer | 158 | 5.3306% | 1948 | 0.7686% |
| C26.9 Ill-defined sites within the digestive system | gastrointestinal malignant tumor | 31 | 1.0459% | 936 | 0.3693% |
| C26.8 Overlapping lesion of digestive system | digestive system tumor | 5 | 0.1687% | 309 | 0.1219% |
| C18-C20 | colorectal cancer | 381 | 12.8543% | 13176 | 5.1990% |
| **Malignant neoplasms of respiratory and intrathoracic organs (C30-C39)** | |  |  |  |  |
| C31 Malignant neoplasm of accessory sinuses | sinus carcinoma | 10 | 0.3374% | 45 | 0.0178% |
| C32 Malignant neoplasm of larynx | laryngeal carcinoma | 22 | 0.7422% | 344 | 0.1357% |
| C32.1 Supraglottis | supraglottic carcinoma | 1 | 0.0337% | 1 | 0.0004% |
| C34.9 Bronchus or lung, unspecified | lung cancer | 1174 | 39.6086% | 59431 | 23.4503% |
| C37 Malignant neoplasm of thymus | thymoma | 9 | 0.3036% | 22 | 0.0087% |
| C38.4 Pleura | pleural malignant tumor | 4 | 0.1350% | 4 | 0.0016% |
| C38.3 Mediastinum, part unspecified | mediastinal neoplasms | 4 | 0.1350% | 33 | 0.0130% |
| **Malignant neoplasms of bone and articular cartilage (C40-C41)** | |  |  |  |  |
| C40.9 Bone and articular cartilage of limb, unspecified | osteocarcinoma | 38 | 1.2821% | 794 | 0.3133% |
| C41.9 Bone and articular cartilage, unspecified | osteosarcoma | 20 | 0.6748% | 403 | 0.1590% |
| **Melanoma and other malignant neoplasms of skin(C43-C44)** | |  |  |  |  |
| C43.9 Malignant melanoma of skin, unspecified | melanoma | 13 | 0.4386% | 14 | 0.0055% |
| C44.1 Skin of eyelid, including canthus | eyelid basal cell carcinoma | 1 | 0.0337% | 1 | 0.0004% |
| C44.3 Skin of other and unspecified parts of face | maxillofacial region carcinoma | 1 | 0.0337% | 272 | 0.1073% |
| C44.9 Malignant neoplasm of skin, unspecified | skin cancer | 1 | 0.0337% | 2 | 0.0008% |
| **Malignant neoplasms of mesothelial and soft tissue(C45-C49)** | |  |  |  |  |
| C49 Malignant neoplasm of other connective and soft tissue | leiomyosarcoma | 6 | 0.2024% | 8 | 0.0032% |
| C49 Malignant neoplasm of other connective and soft tissue | sarcoma (including synovial sarcoma, liomyosarcoma、tongue angiosarcoma, rhabdomyosarcoma, soft tissue sarcoma, retroperitoneal sarcoma, liposarcoma, fibrosarcoma） | 20 | 0.6748% | 39 | 0.0154% |
| **C50 Malignant neoplasm of breast** | breast cancer | 602 | 20.3104% | 17507 | 6.9079% |
| **Malignant neoplasms of female genital organs(C51-C58)** | |  |  |  |  |
| C52 Malignant neoplasm of vagina | vaginal carcinoma | 3 | 0.1012% | 7 | 0.0028% |
| C53.9 Cervix uteri, unspecified | cervical cancer | 156 | 5.2632% | 6707 | 2.6464% |
| C54.1 Endometrium | endometrial carcinoma | 32 | 1.0796% | 373 | 0.1472% |
| C54.9 Corpus uteri, unspecified | uterine cancer | 4 | 0.1350% | 42 | 0.0166% |
| C55： Malignant neoplasm of uterus, part unspecified | metrocarcinoma | 11 | 0.3711% | 100 | 0.0395% |
| C56 Malignant neoplasm of ovary | ovarian cancer | 190 | 6.4103% | 3028 | 1.1948% |
| C56 Malignant neoplasm of ovary | malignant germ cell tumors | 2 | 0.0675% | 6 | 0.0024% |
| C57.0 Fallopian tube | fallopian tube carcinoma | 4 | 0.1350% | 23 | 0.0091% |
| C57.9 Female genital organ, unspecified | gynecological tumor | 5 | 0.1687% | 158 | 0.0623% |
| C57.9 Female genital organ, unspecified | gestational trophoblastic tumor | 1 | 0.0337% | 12 | 0.0047% |
| C58 Malignant neoplasm of placenta | choriocarcinoma | 4 | 0.1350% | 44 | 0.0174% |
| **Malignant neoplasms of male genital organs(C60-C63)** | |  |  |  |  |
| C61 Malignant neoplasm of prostate | prostate cancer | 53 | 1.7881% | 586 | 0.2312% |
| C62.9 Testis, unspecified | testicular cancer | 18 | 0.6073% | 53 | 0.0209% |
| C62.9 Testis, unspecified | seminoma | 9 | 0.3036% | 34 | 0.0134% |
| **Malignant neoplasms of urinary tract(C64-C68)** | |  |  |  |  |
| C64 Malignant neoplasm of kidney, except renal pelvis | renal carcinoma | 38 | 1.2821% | 107 | 0.0422% |
| C66 Malignant neoplasm of ureter | ureteral carcinoma | 1 | 0.0337% | 1 | 0.0004% |
| C67 Malignant neoplasm of bladder | bladder cancer | 43 | 1.4507% | 419 | 0.1653% |
| **Malignant neoplasms of eye, brain and other parts of central nervous system (C69-C72)** | |  |  |  |  |
| C71 Malignant neoplasm of brain | brain cancer | 31 | 1.0459% | 655 | 0.2584% |
| C71.9 Brain, unspecified | primitive neuroectodermal tumor | 3 | 0.1012% | 2 | 0.0008% |
| **Malignant neoplasms of thyroid and other endocrine glands (C73-C75)** | |  |  |  |  |
| C73 Malignant neoplasm of thyroid gland | thyroid carcinoma | 12 | 0.4049% | 19 | 0.0075% |
| C74 Malignant neoplasm of adrenal gland | adrenal carcinoma | 2 | 0.0675% | 2 | 0.0008% |
| C75.1 Pituitary gland | pituitary adenoma | 1 | 0.0337% | 2 | 0.0008% |
| **Malignant neoplasms of ill-defined, secondary and unspecified sites (C76-C80)** | |  |  |  |  |
| C76.1 Thorax | chest wall carcinelcosis | 2 | 0.0675% | 4 | 0.0016% |
| C76.2 Abdomen | malignant abdominal tumors | 7 | 0.2362% | 19 | 0.0075% |
| C76.2 Abdomen | abdominal wall cancer | 2 | 0.0675% | 2 | 0.0008% |
| **Malignant neoplasms, stated or presumed to be primary, of lymphoid, haematopoietic and related tissue (C81-C96)** | |  |  |  |  |
| C82.9 Follicular lymphoma, unspecified | lymphosarcoma | 2 | 0.0675% | 4 | 0.0016% |
| C90.0 Multiple myeloma | multiple myeloma | 44 | 1.4845% | 684 | 0.2699% |
| C95.9 Leukaemia, unspecified | leukemia | 92 | 3.1039% | 5081 | 2.0049% |
| **C96 Other and unspecified malignant neoplasms of lymphoid, haematopoietic and related tissue** | |  |  |  |  |
| C96.9 Malignant neoplasm of lymphoid, haematopoietic and related tissue, unspecified | lymphoma | 227 | 7.6586% | 2607 | 1.0287% |
| **C96.8 Histiocytic sarcoma** | malignant histiocyte carcinama | 1 | 0.0337% | 1 | 0.0004% |
| **O01 Hydatidiform mole** | hydatidiform mole | 5 | 0.1687% | 140 | 0.0552% |
| **precancerous conditions*** | |  |  |  |  |
|  | chronic atrophic gastritis（gastric precancerous lesion） | 80 | 2.6991% | 10094 | 3.9829% |
|  | esophageal precancerous lesion | 1 | 0.0337% | 200 | 0.0789% |
|  | colon precancerous lesion | 3 | 0.1012% | 380 | 0.1499% |
|  | cervical precancerous lesion | 10 | 0.3374% | 905 | 0.3571% |
|  | myelodysplastic syndrome(Leukemia precancerous lesion) | 2 | 0.0675% | 87 | 0.0343% |
|  | laryngeal precancerous lesion | 1 | 0.0337% | 33 | 0.0130% |
|  | hepatic precancerous lesion | 1 | 0.0337% | 60 | 0.0237% |
| **not reported** |  | 92 | 3.1039% | 310 | 0.1223% |
| **Total** |  | 2964 | 100.0000% | 253,434 | 100.0000% |

* no ICD-10 code available
