# Supplementary material for: Traditional Chinese Medicine in Cancer Care: A Review of Controlled Clinical Studies Published in Chinese
Source: PLoS One. 2013 Apr 3;8(4):e60338. doi: 10.1371/journal.pone.0060338 (PMC3616129; doi:10.1371/journal.pone.0060338)
Supplement: Table S3 — Characteristics of studies with significant evidence of TCM treatments for cancer published in Chinese. The characteristics of 5 RCTs of TCM for cancer reporting randomization methods and blinding information with outcomes of survival, relapse and/or metastasis and quality of life. (DOC) [file pone.0060338.s003.doc]

Table S3 Characteristics of studies with significant evidence of TCM treatments for cancer published in Chinese (5 papers)

| study ID | funding | participants (No.) | randomization method | blinding to whom | intervention VS control | outcomes | main results | recommendation for generalization stated in the paper (yes/no) |
| --- | --- | --- | --- | --- | --- | --- | --- | --- |
| Huang S 2011 | not reported | acute leukemia (20 VS 21) | computer software | 12 | Fufang Zhebei Granule + chemotherapy VS chemotherapy + Placebo | survival, relapse, clinical symptom, laboratory indexes, relief time | The median survival time did not show significant difference between treatment and control group, but the relapse rate in 3 months, 6 months and 1 year was lower in treatment group than in control group. (p<0.05) | no |
| Xu ZY 2007 | Science Technology Development Funding of Shanghai City | advanced non-small cell lung cancer (60 VS 56) | envelope | 34 | Kangliu Zengxiao Fang + Yan Ning Decoction + chemotherapy VS chemotherapy | survival, quality of life, clinical symptoms, side effects | the median survival time in treatment group was 15.57 months, and in control group 11.17 months (p<0.01), treatment group showed higher quality of life after treatment (p<0.01) | no |
| Wang LF 2010 | Key Program of Science Development of Shanghai City | advanced lung cancer (44 VS 46) | random table | single blinding | Yan Ning Decoction + chemotherapy VS chemotherapy | tumor size, survival, quality of life, clinical symptom, side effects | median survival: treatment group 16.63 months, control group 11.60 months (p<0.05), and treatment group showed higher survival rate for 1 year, 3 year and 5 years (p<0.05), and better improvement of quality of life (p<0.05) and clinical symptoms (p<0.05) | no |
| Liu HT 2009 | not reported | breast cancer (200 VS 200 VS 200) | SAS computer software | patients, physicians | treatment I group (Jianpi Yishen Capsule VS treatment II group (Yangyin Shengxue Mixture) VS control group (placebo) | TCM syndrome related symptoms, quality of life, immune function, relapse and metastasis, survival and survival time without disease | two herbal medicine showed significant improvement of clinical symptoms, quality of life, immune function and one year, two year survival | yes |
| LI LN 2003 | not reported | non-small cell lung cancer (40 VS 40 VS 40) | computer software | 1 | TCM group (individualized decoction based on syndrome differentiation + herbal injection + standard herbal decoction + routine treatment) VS Conventinal medicine group (chemotherapy + herbal decoction placebo + routine treatment) VS integrative group (individualized decoction based on syndrome differentiation + herbal injection + standard herbal decoction +chemotherapy + routine treatment) | survival time | TCM group: 84.4% 0.5 year survival and 18.1% 1 year survival; conventional medicine group: 82.5% 0.5 year survival and 20.6% 1 year survival; intergrative group: 92.5% 0.5 year survival and 42.7% 1 year survival (p<0.05) | yes |
